# From Benign to Malign in a Case of Cervical Adenopathy in a 17-Year-Old Adolescent: Diagnostic Traps

**DOI:** 10.1155/2016/5173849

**Published:** 2016-11-27

**Authors:** Simona Dumitra, Maria Trailescu, Amelia Burlea, Claudia Covaci, Ozana Balan, Adrian Pavel, Carmen Crișan

**Affiliations:** Vasile Goldiș Western University of Arad, Arad, Romania

## Abstract

Distinguishing between benign and malign adenopathies remains a challenge and could represent a source of error in a diagnosis. We report a case of right laterocervical adenopathy in a 17-year-old teenager admitted to hospital with an episode of fever associated with dysphagia, congested pharynx, and pultaceous deposits. Initially the adenopathy was considered to be secondary to a coinfection with* Streptococcus* B-hemolytic and Epstein-Barr virus, as suggested by the positive bacteriological and serological tests. The onset of the adenopathy before the episode and the ultrasound modifications raised the suspicion of a malignancy, later confirmed by the histopathologic examination of the lymph node excision. The final diagnosis was nodal metastasis of an undifferentiated lymphoepithelial carcinoma with an ENT starting point. Currently, the adolescent is hospitalised in the ENT ward, where the pharynx carcinoma with nodal metastasis was confirmed. Sometimes the infectious context can mask or unmask a malign chronic disease with insidious evolution.

## 1. Introduction

Cervical adenopathy remains a current problem in pediatrics. Although most of the cases have an infectious or benign etiology, it is important for the doctor to be vigilant and to exclude the rare cases or the neoplastic diseases, for a prompt diagnosis and optimum therapeutic decision.

The etiology of cervical adenopathies is complex, varying between benign causes, malign causes, and nodal metastasis. Benign causes include infections such as Epstein-Barr virus, CMV, atypical mycobacteria, TBC, HIV, and* Bartonella henselae*. Malign causes include leukemias, lymphomas, neuroblastoma, and rhabdomyosarcoma. Lastly, nodal metastasis of nasopharyngeal carcinoma, thyroid cancer, or parathyroid tumors can occur [1–9].

The lymphoepithelial carcinoma of the pharynx is a malignity that is histologically characterised by an undifferentiated carcinoma with intermixed reactive lymphoplasmacytic infiltrate, seldom encountered in childhood [10].

Rapid elucidation of the etiology allows a treatment with optimum effect. In addition, the benign/malign differentiation allows an adequate attitude and decision taking that can extend the patient's life.

## 2. Case Report

**Figure 1:**
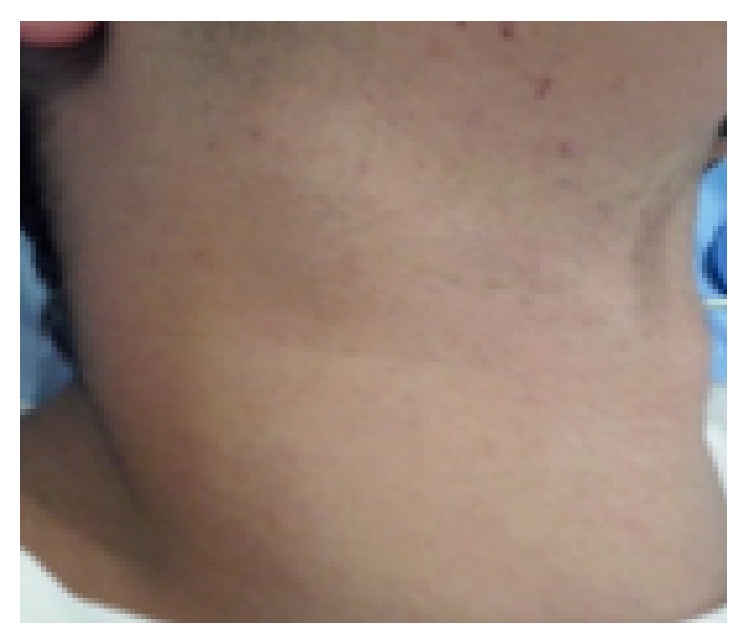
Clinical aspect.

FLE 17-year-old male from an urban environment is admitted to hospital in September 2015 for fever, unwellness, and dysphagia. The symptoms appeared 3 days before admission with febrile seizures of 39-40°C, dysphagia, and food refusal. The clinical examination showed a teenager with proper nutrition, anterior cervical adenopathy 44/40 mm, tender, painful, mobile, and nonadherent to the deep planes, no signs of local inflammation (Figure 1), hypertrophic tonsils, and pultaceous deposits.

The results of the laboratory tests are as follows: ESR, 35/50 mm at 1/2 h; C-reactive protein, 10 mg/dL (normal 0.5 mg/dL); ASLO, 472 UI/mL; LDH, 147 U/I (normal 125–220 U/I); transaminases and direct bilirubin, normal; positive pharyngeal exudate for beta-hemolytic group A* Streptococcus*; EBV-VCA IgM Ab, positive; EBV-VCA IgG Ab, positive; tests for CMV, toxoplasmosis, HIV,* Bartonella henselae*, and syphilis, negative; Quantiferon TB Gold Test, negative.

The thoracic X-ray, abdominal ultrasound, and color Doppler echocardiography were all normal.

The first ENT examination revealed pseudomembranous tonsillitis. Therefore, infectious mononucleosis was suspected.

**Figure 2:**
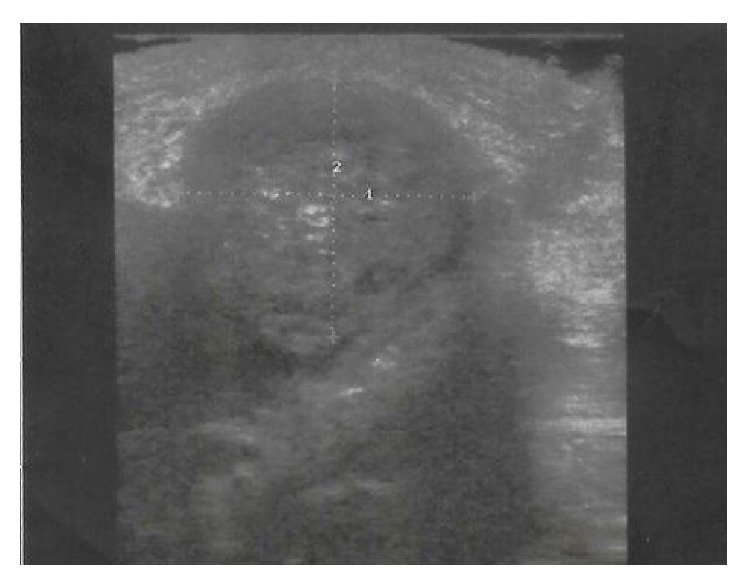
The ultrasound performed, gray scale mode B of the cervical mass. Solbiati index is L/T, respectively, *D*2/*D*1 = 23.6 mm/26.9 mm = 0.87.

The ultrasound, gray scale mode B, of the cervical mass showed a well-defined adenopathy 23.6 mm/26.9 mm in size, corresponding to a Solbiati index of 0.87. The lymph node is round, well defined, hypoechoic, and with intranodal calcification and no echogenic hilus (Figure 2).

The correlation between the clinical and laboratory data for the actual episode raised the suspicion of a coinfection with Beta-hemolytic* Streptococcus* and Epstein-Barr virus. Hence, the patient was given penicillin for 10 days. This led to an obvious improvement of the symptoms as follows: the fever subsided in 5 days and the dysphagia in 3 days, the pultaceous deposits disappeared in 4 days, and the adenopathy was reduced to 30/29 mm.

The previous history of the adenopathy that appeared 6 months before the infectious episode with uneven evolution and malignancy characteristics required a lymph node biopsy, as highlighted by the lymph node ultrasound.

**Figure 3:**
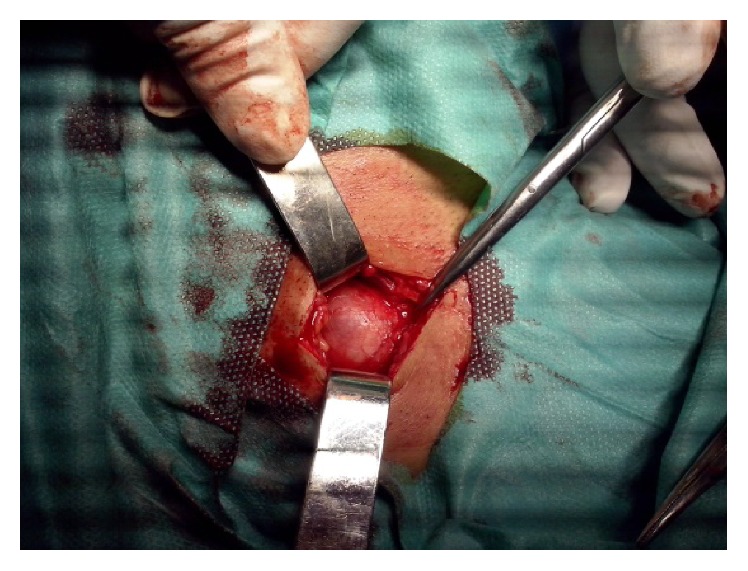
Intraoperative aspects.

**Figure 4:**
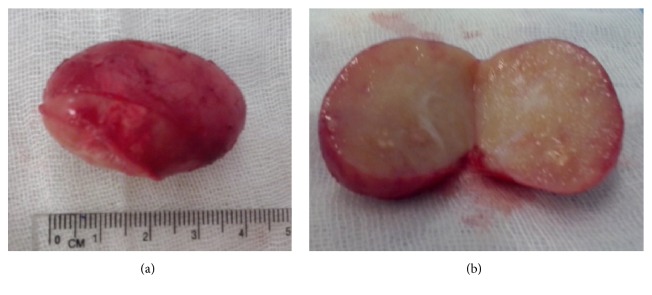
Macroscopic aspects.

Figure 3 shows the intraoperative aspects during lymph node excision. The following observations were made: enlarged right laterocervical lymph node and hard, well-encapsulated, no “sentinel” lymph nodes, adherent to the supra- and subjacent planes with the section aspect of “fish meat,” malignity macroscopic aspect (Figures 4(a) and 4(b)).

The histopathologic examination suggests the presence of a lymph node metastasis of an undifferentiated lymphoepithelial carcinoma with an ENT starting point. Currently, the teenager is hospitalised in the ENT ward, where the diagnosis of pharynx carcinoma with nodal metastasis was confirmed by endoscopy and biopsy. No evidence of metastasis in other areas was found. The thoracic and abdominal MRI was normal.

## 3. Discussions

The apparent acute evolution, the onset with high fever, dysphagia, pseudomembranous tonsillitis, the right laterocervical location, and the favourable response to the antibiotic treatment initially suggested an infectious etiology with intricate elements of streptococcal and infectious mononucleosis. No hepatosplenomegaly was detected and no associated adenopathies were recorded.

The elements that suggested malignity were the onset of the adenopathy 6 months before, the persistence and the ultrasound aspect of the cervical mass, round shape, Solbiati index lowered below 2, the absence of the echogenic hilus, the aspect of calcification inside the lymph node, and the hypoechoic mass with hyperechoic echoes. The Solbiati index (SI) represents the ratio of the largest to the smallest diameter. Values above 2 are associated with a benign process, whereas values below 2 are correlated with malignancy [11].

Due to all these elements (Table 1) a lymph node biopsy was required. The histopathologic examination confirmed the malignity of the adenopathy.

Lymphoepithelial carcinoma is a rare malignancy in children. Rytkönen et al. [10] in a meta-analysis of the PubMed articles spanning more than 30 years identified 110 cases in ENT, of which 19 were at the oropharynx level. None of these cases was of a child, with the average age of the cases being 58–62 years.

As a result, being able to distinguish between benign and malign adenopathies is of great interest to the clinician.

Yaris et al. [12] analysed a group of 126 pediatrics patients with cervical adenopathies; they noted that the main alarming elements for malignity are greater age, supraclavicular lymph nodes, dimensions above 3 cm, generalised adenopathies, hepatosplenomegaly, the presence of mediastinal masses, and increased LDH values.

Al Kadah et al. in 2015 [13] performed a study on 251 patients and found the following statistically significant factors associated with the cervical mass neoplasia: generalised lymphadenopathy, history of malign diseases, adherent cervical mass, immobile, increased diameter, bulky lesions, absence of hilus, irregular external shape, the protective role of the long forms and low Solbiati index values in the ultrasound B mode gray scale examination and laboratory parameters for thrombocytopenia, and high CRP and LDL. The persistence of a cervical adenopathy for more than 3 weeks warrants a biopsy.

Asai et al. [14] have created a score to differentiate between the benign and malign cervical lymphadenitis based on ultrasound. This includes 7 criteria: the ratio between the largest and smallest diameter under 2, multiple lymph nodes, fusion tendency, irregular edges, hypoechoic masses with intense hyperechoic echoes, presence of line echoes, and absence of hilus. The presence of 3 or more of these criteria suggests 98% malignancy.

Ahuja and Ying [15] highlight that the malignancy elements in the cervical adenopathies are the round shape, absence of hilus, necrosis inside the lymph node, reticulated appearance, calcifications, matting, subcutaneous cellular tissue edema, and peripheric vascularization.

Kanamori et al. [16] and Poantă et al. [17] suggested that the use of CEUS in distinguishing between benign and malign cervical adenopathies is superior compared with gray scale B mode and Doppler ultrasound. In addition, it must be used to remove the erroneous interpretations resulting from these techniques.

## 4. Conclusions

The streptococcal and Epstein-Barr coinfection with the “noisy” clinical manifestation unmasked the insidious evolution of a malign lymphoepithelioma (as confirmed by the histopathologic examination of the lymph node biopsy) with a pharyngeal starting point, a very rare condition in children. However, it could have resulted in a delay of the primary diagnosis by interpreting the cervical mass in the infectious context.

The lymph node ultrasound is a useful element that can raise malignancy suspicions even in an apparent benign clinical context.

## Figures and Tables

**Table 1:** Benign versus malign criteria for the reported case.

Criteria pleading for benign cervical mass	Criteria pleading for malign cervical mass
Anterior cervical location	The onset of the adenopathy 6 months before the infectious process
*Streptococcus* and Epstein-Barr coinfection context	Solbiati index below 2
Reduction of the adenopathy under antibiotic treatment	Intranodal calcification
The absence of any symptoms after resolving the acute process	No echogenic hilus
Normalisation of the inflammatory markers after the tonsillitis bout	Persistence of the adenopathy
Absence of any malignity signs at the first ENT control	Hypoechoic mass with hyperechoic echoes
Normal values of LDH and complete blood count	Round shape
Well-differentiated lymph node edges as revealed by ultrasound	
